# Polyclonal Aptamers for Specific Fluorescence Labeling and Quantification of the Health Relevant Human Gut Bacterium *Parabacteroides distasonis*

**DOI:** 10.3390/microorganisms9112284

**Published:** 2021-11-02

**Authors:** Hu Xing, Ann-Kathrin Kissmann, Heinz Fabian Raber, Markus Krämer, Valerie Amann, Kathrin Kohn, Tanja Weil, Frank Rosenau

**Affiliations:** 1 Institute for Pharmaceutical Biotechnology, Ulm University, Albert-Einstein-Allee 11, 89081 Ulm, Germany; dolphinzyt2418@gmail.com (H.X.); ann-kathrin.kissmann@uni-ulm.de (A.-K.K.); Heinz.Raber@uni-ulm.de (H.F.R.); Markus-1.Kraemer@uni-ulm.de (M.K.); Valerie.amann@uni-ulm.de (V.A.); kathrin.kohn@uni-ul.de (K.K.); 2 Max Planck Institute for Polymer Research Mainz, Ackermannweg 10, 55128 Mainz, Germany; weil@mpip-mainz.mpg.de

**Keywords:** aptamer, *Parabacteroides distasonis*, FluCell-SELEX, biosensor development

## Abstract

Single-stranded DNA aptamers as affinity molecules for the rapid, reliable detection of intestinal bacteria are of particular interest to equip health systems with novel robust and cheap diagnostic tools for monitoring the success of supplementation strategies with selected probiotic gut bacteria in the fight against major widespread threats, such as obesity and neurodegenerative diseases. The human gut bacterium *Parabacteroides distasonis* (*P. distasonis*) is positively associated with diseases such as obesity, non-alcoholic fatty liver disease and multiple sclerosis with reduced cell counts in these diseases and is thus a promising potential probiotic bacterium for future microbial supplementation. In this paper we report on the evolution of a specific polyclonal aptamer library by the fluorescence based FluCell-SELEX directed against whole cells of *P. distasonis* that specifically and efficiently binds and labels *P. distasonis*. The aptamer library showed high binding affinity and was suited to quantitatively discriminate *P. distasonis* from other prominent gut bacteria also in mixtures. We believe that this library against a promising probiotic bacterium as a prototype may open new routes towards the development of novel biosensors for the easy and efficient quantitative monitoring of microbial abundance in human microbiomes in general.

## 1. Introduction

*Parabacteroides distasonis* (*P. distasonis*) has been described as a Gram-negative, obligately anaerobic, non-spore-forming, non-motile, and rod-shaped bacterium [1]. This intestinal microbe plays a vital role in the health of the human body. Recent years’ research has clearly demonstrated that an imbalance of *P. distasonis* as part of a dysbiosis in the human gut microbiome is associated with a variety of diseases, including obesity, nonalcoholic fatty liver, multiple sclerosis and the development of colon tumors [2,3,4,5]. With the help of bacterial 16S rRNA gene sequencing technique Chen et al. have stated that abnormalities in the intestinal flora diversity revealed a significant reduction of *Bacteroidetes* species in the intestine of cirrhotic patients compared to normal subjects [6]. In a study on *P. distasonis* and obesity-related mouse models, Wang *et al*. found that oral administration of living *P. distasonis* led to a reduction in body weight and improved glucose homeostasis in the obese group of mice while correcting abnormal symptoms associated with obesity, including hyperlipidemia and hepatic steatosis [7]. De Boer et al. found that the introduction of *P. distasonis* into obese mice greatly improved the distribution of secondary bile acids in mice, which further affected lipid metabolism in mice [8]. In another study, Pfalzer et al. described a negative correlation between *P. distasonis* and intestinal tumor burden in Apc1638N mice [9]. It was further demonstrated that feeding freeze-dried preparations of *P. distasonis* to mice inhibited AOM and high-fat diet-driven colon tumor formation [10]. As *P. distasonis* has a wide range of potential applications in disease prevention, as a widely accepted intestinal symbiotic and beneficial bacterial species *P. distasonis* could be used as a promising novel healthy probiotic strain for regulating host metabolism to reduce obesity and relieve diseases such as metabolic dysfunction. As the abundance of *P. distasonis* in the human gut is closely correlated with the occurrence of several diseases, the rapid analysis of gut microbiome composition has become a task to be urgently solved, as traditional analyses including DNA sequencing and quantitative PCR are not only time-consuming, but also cumbersome to achieve a rapid detection [11].

Modern biosensors are characterized by high selectivity towards their dedicated analyte, reproducibility, stability, sensitivity, and linearity [12]. They depend on combinations of optical or electronic read out techniques with high-affinity biomolecules and aim on allowing for specific and sensitive binding of various analytes to the sensor. This poses a pivotal role especially to these binding entities responsible for the “bioaffinity” of sensor devices. Today the majority of such bioaffinity molecules for the development of biosensors still are antibodies or their derivatives, but this dominant position is now increasingly being taken over by aptamers [13]. Nucleic acid-based aptamers are single-stranded RNA or short DNA sequences with, compared to antibodies, advantageous physico-chemical properties, such as high stability, high affinity also for small molecules and low molecular sizes [14]. In addition, they are easy to modify, easy to synthesize in vitro, and allow for flexible sequence design [15]. These inherent advantages not only facilitate target diversification but also lead to a significant increase in detection efficiency due to the high efficiency and excellent binding constants. For all these reasons, aptamers have been emerged as promising novel recognition elements in the biosensor field [15,16].

In 1995, Eaton et al. first argued that oligonucleotide aptamer libraries have a significantly higher affinity for protein molecules than small molecule targets or conventional peptide combinatorial libraries [17,18]. Aptamers have been pioneered in the field of medical detection and diagnostics, and in 2016 Kumar et al. used aptamers to specifically recognize viral surface proteins and monitor intact viruses [19], and aptamers can also be used as a probe to specifically detect various cancer cells, such as liver cancer [20], breast cancer [21,22] or lung carcinoma [23]. 

Usually, aptamers are isolated in vitro from synthetic oligonucleotide libraries using a technique called SELEX (Systematic Evolution of Ligands by Exponential Enrichment), combined with PCR amplification in vitro to exponentially enrich oligonucleotides that bind specifically to target molecules, and after repeated screening and amplification, the final aptamer is obtained with high specificity and affinity to the target based on spatial structure. Since its first application in 1990, SELEX technology has undergone several modifications and developments to make the selection process more efficient [24]. Variants have been developed like the high-throughput sequencing SELEX [25], in vivo SELEX [26], cell SELEX [27], and Microfluidic SELEX [28,29]. For this reason, these updated SELEX methods now enable the simple isolation of fully functional aptamers from highly complex mixtures such as plasma proteins and cell surfaces.

Recently, we have developed the FluCell-SELEX [13], which is conceptually based on the FluMag SELEX [30] and uses Cyanine 5 (Cy5) fluorescently labeled aptamer libraries to monitor the evolution process of the binding properties towards target microbial cells [13].

**Figure 1:**
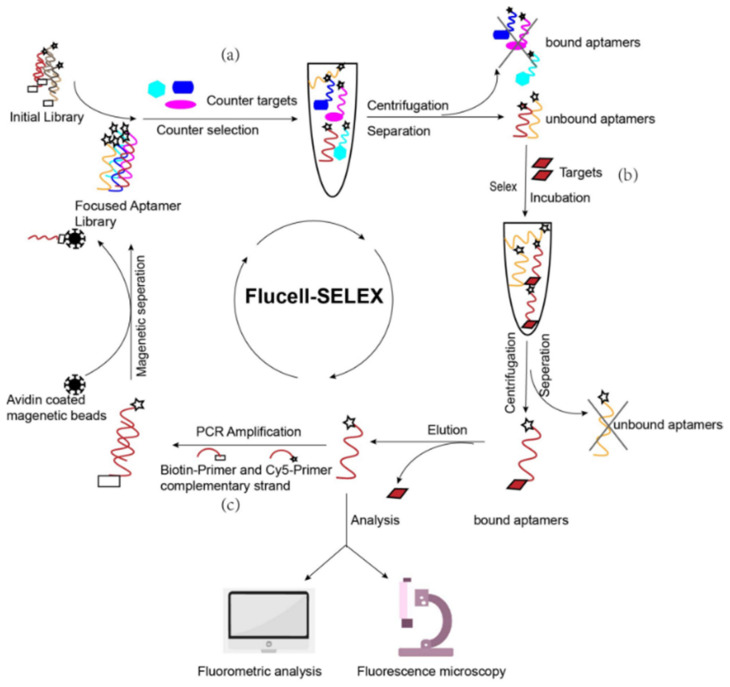
Experimental set-up of this study: Selection of polyclonal aptamer libraries against *P. distasonis* using the FluCell-SELEX. Including (**a**) Counter selection, in which the randomized initial single stranded aptamer library is incubated with bacteria mixtures containing *A. muciniphila*, *A. stercoricanis*, *B. producta*, *R. microfusus* and *R. intestinalis* to increase the selection pressure and to raise the stringency during the SELEX process. Aptamers bound to negative cells can be removed by simple washing through this process. (**b**) Target selection. The non-binder aptamers from the counter selection are further incubated with the target cell to screen for aptamer sub-populations that bind specifically to the target cell. Aptamers that do not bind to the target cell are removed by washing. (**c**) Enrichment of the library. Aptamers bound to the target cell are amplified by PCR after elution and provided with the fluorescent Cy5-label introduced via the Cy5-primer and biotin-primer and subsequently separated with avidin coated magnetic beads to obtain the focused library. This screening process is repeated to obtain a polyclonal library that specifically binds and labels the target cells fluorescently. The specificity of this aptamer library was subsequently analyzed by modern bioanalytical techniques, including fluorometric analysis and fluorescence microscopy.

Here we describe the evolution of an aptamer library that specifically can recognize *P. distasonis* by the FluCell-SELEX (Figure 1). The library evolution process was monitored by fluorescence analysis to accurately measure the binding of aptamers to the target cells. An initial library was composed of 10^12^–10^16^ individual aptamers with 40 randomized bases flanked by two primer binding sites (23 nt each). Inspired by Liu et al. the screening process involved rigorous counter selections [31], in this study using mixtures of other high abundant gut bacteria containing *Akkermannsia muciniphila, Allobaculum stercoricanis, Blautia producta, Rikenella microfusus* and *Roseburia intestinalis* to increase the selection pressure and to raise stringency during the SELEX process. The final Cy5-labeled aptamer library was then further analyzed in detail regarding its ability to specifically label target cells for their discrimination in fluorescence microscopy and in mixtures with the control gut bacteria in quantitative fluorometric microtiter plate assays.

## 2. Materials and Methods

### 2.1. Cell Lines and Cell Culture

The bacteria strains *P. distasonis* (DSM-29491), *A. muciniphila* mucT (DSM-22959), *A. stercoricanis* (DSM-13633), *R. intestinalis* (DSM-14610), *B. producta* (DSM-29491), and *R. microfusus* (DSM-15922) were cultivated in Schaedler Broth Medium (Carl Roth, Karlsruhe, Germany) at 37 °C under anaerobic conditions. 

### 2.2. Single-Stranded DNA (ssDNA) Library and Primers

The random sequence library was synthesized and purified (TriLink BioTechnologies, Inc., San Diego, CA, USA). The sequence was as follows: 5′-TAGGGAAGAGAAGGACATATGAT-N (40) -TTGACTAGTACATGACCACTTGA-3′. The initial library consisted of three parts. The random part contained a randomly varying central region consisting of 40 nucleotides and two fixed sequences of 23 nucleotides each, which could be specifically and complementarily bound to the primers. Cyanine 5-labeled forward primer (Cy5-P1) 5′-Cy5-TAGGGAAGAGAAGGACATATGAT-3′, and biotin-labeled reverse primer (biotin-P2) 5′-biotin-TCAAGTGGTCATGTACTAGTCAA-3′ (Eurofins Genomics, Ebersberg, Germany) [13].

### 2.3. Cell-SELEX

Cell-SELEX included counter SELEX as well as target SELEX. In this chapter, the non-target bacteria used in counter SELEX were *A. muciniphila*, *A. stercoricanis*, *R. intestinalis*, *B. producta*, and *R. microfusus*. Aptamers that did not bind to negative bacteria were obtained, which could be further screened by target SELEX to gain an aptamer library specifically targeting *P. distasonis*. The Cell-SELEX could be divided into the following steps and were carried out according to the FluCell-SELEX method by Kubiczek et al.

#### 2.3.1. Cell Pretreatment

The cells were incubated under anaerobic conditions for 21–24 h and subsequently centrifuged at 7500× *g* for 2 min and then washed 3 times with 1× DPBS buffer (Thermo Fisher Scientific, Waltham, MA, USA). Finally, the OD_600_ of the bacterial solution was adjusted to 1.

#### 2.3.2. Aptamer Library Activation

0.5 nmol of the original library or 10/5 pmol ssDNA library was added to 500 μL 1× DPBS, and incubated at 95 °C for 5 min, ice bath for 5 min, and then at room temperature for 20 min to ensure that the aptamers have the same 3D structure.

#### 2.3.3. Screening

BSA (100 mg/mL) (Sigma Aldrich (St. Louis, MO, USA) and tRNA (10 mg/mL) (Sigma Aldrich (St. Louis, MO, USA) with increasing stringent were added to the activated ssDNA library to reduce non-specific adsorption. The activated library was incubated with negative cells for 1h at 37 °C and centrifuged at 3000× *g* for 2 min. The aptamer library was then incubated with *P. distasonis* at 37 °C. The supernatant was then removed by centrifugation at 3000× *g* for 2 min and finally, the pellet was washed with 1× DPBS to discard unbound nucleic acids (see Table S1).

#### 2.3.4. Elution

Cells from the previous step were resuspended in 100 μL 1× DPBS and incubated at 95 °C for 5 min to disrupt the aptamer 3D structure and separate the cells from the aptamer. The aptamer bound to *P. distasonis* was collected by subsequent centrifugation at 11,000× *g* for 2 min.

#### 2.3.5. Acquisition of Secondary Libraries

The collected aptamers were amplified by PCR. The amplification conditions were as follows: 2 min at 80 °C, 2 min at 85 °C, 2 min at 90 °C, 3 min at 94 °C, followed by 20 cycles of 30 sec at 94 °C, 30 sec at 56 °C, 30 sec at 72 °C, then 2 min at 72 °C. The PCR product was subsequently bound to the streptavidin labeled magnetic beads (Qiagen, Hilden, Germany). Following denaturation in 0.1 M NaOH, the single-stranded DNA was isolated, collected, and refold the new DNA pool for the next selection round.

#### 2.3.6. Binding Assay

*P. distasonis* was preprocessed by the above method (see Section 2.3.1). The binding affinity of the aptamer library was determined by incubating *P. distasonis* (1 mL OD_600_ = 1) with 5 pmol of activated Cy5-labeled aptamer library in 500 µL of 1× DPBS for 30 min at 37 °C. Subsequently, the supernatant was removed by centrifugation at 3000× *g* for 2 min, and the pellet was resuspended in 100 μL of 1× DPBS buffer after washing three times to obtain cell-bound aptamers after elution. Finally, the fluorescence intensity to determine the screening status was measured using an Infinite M200 spectrophotometer (TECAN, Männedorf, Switzerland) at an excitation wavelength of 637 nm and an emission of 670 nm.

### 2.4. Determination of High Specificity Aptamer Libraries

#### 2.4.1. Specificity Analysis

The bacterial solution of *P. distasonis*, *A. muciniphila*, *A. stercoricanis*, *R. intestinalis*, *B. producta*, and *R. microfusus* were preprocessed by the above methods (see Section 2.3.1). The specificity analysis of the aptamer library was determined by incubating each bacterium (1 mL OD_600_ = 1) with 10 pmol of activated Cy5-labeled aptamer library in 500 µL of 1× DPBS for 30 min at 37 °C. Each group of samples was postprocessed as described above (see Section 2.3.6). Finally, the specificity of the aptamer library was analyzed by comparing the fluorescence intensity of each experimental group and PBS as the negative control group.

#### 2.4.2. Affinity Analysis

The bacterial solution of *P. distasonis* was preprocessed by the above method (see Section 2.3.1). The binding affinity of the selected aptamer library was determined by incubating *P. distasonis* (1 mL OD_600_ = 1) with varying concentrations of aptamer candidates in 500 µL of 1× DPBS for 30 min at 37 °C. Finally, the dissociation constants (K_d_) of the fluorescent aptamers were determined by fitting the dependence of the fluorescence intensity on the concentration of the aptamers to the equation Y = B_max_X/(K_d_ + X) using Graphpad Prism 8 (GraphPad Software, San Diego, CA, USA).

#### 2.4.3. Fluorescence Microscopy

The bacterial solution of *P. distasonis*, *A. muciniphila*, *A. stercoricanis*, *R. intestinalis*, *B. producta*, and *R. microfusus* were pre-treated by the above method (see Section 2.3.1). 20 pmol of the aptamer library in 500 µL of 1× DPBS were activated as described earlier (see Section 2.3.2). Subsequently the libraries were incubated with each bacterium (1 mL OD_600_ = 1) for 30 min at 37 °C. After centrifugation at 3000× *g* for 2 min, the supernatant was removed. The pellet was washed once with 500 µL of 1× DPBS and then resuspend in 500 µL of 1× DPBS. Afterward, 100 µL of each bacterial mixture was transferred to a 96-well microplate. Finally, the fluorescence imaging of each group was obtained using fluorescence microscopy, which was performed using a Leica DMi8 coded (Leica Microsystems CMS GmbH, Wetzlar, Germany) at ×20 magnitude under transmitted light with the Y5 filter (excitation: 590–650 nm and emission: 662–738 nm).

## 3. Results

**Figure 2:**
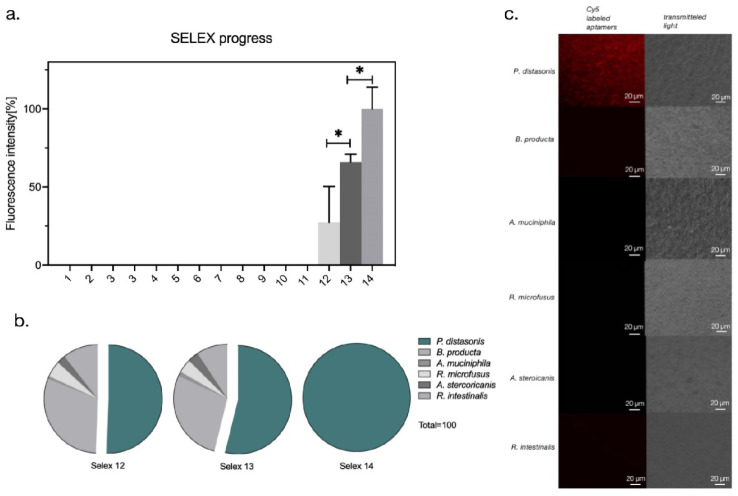
Binding and specificity analysis of polyclonal aptamer library selectively targeting *P. distasonis*. (**a**) Increased fluorescence intensity indicate the enrichment of specific Cy5-labeled aptamers binding to *P. distasonis* at equal concentrations and washing three times with 1× DPBS before elution; *p-*values < 0.05 were considered significant; * denotes *p* < 0.05. (**b**) Increased specificity of the polyclonal aptamer library binding to *P. distasonis* compared to other five gut bacteria *B. producta*, *A. muciniphila*, *R. microfusus*, *A. stercoricanis*, and *R. intestinalis*. All experiments were performed with 1 mL cells with OD_600_ of 1 and 10 pmol aptamers in triplicates. The fluorescence intensity was measured at an excitation of 635 nm and an emission of 670 nm. (**c**) Fluorescence microscopy of Cy5-labeled polyclonal aptamer library R14 labeled with *P. distasonis*. The binding of the fluorescently labeled polyclonal aptamer library to *P. distasonis* showed a strong fluorescent signal. The other five intestinal bacteria *B. producta*, *A. muciniphila*, *R. microfusus*, *A. stercoricanis*, and *R. intestinalis* served as negative controls.

A total of fourteen rounds of the FluCell-SELEX were performed with counterselections against control bacteria (or mixtures of them) according to the conditions given in Table S1. In the fluorescent binding assays for testing the labeling of *P. distasonis* cells by measuring the fluorescence intensity of the aptamers bound to a constant number of cells it became obvious that the ability of the aptamer libraries to recognize *P. distasonis* was significantly enhanced after 14 rounds of screening to *P. distasonis*. Interestingly the initial labeling efficiency developed slowly with fluorescence levels resulting from binding of the libraries from round 1 to 11 essentially being below the detection limit of the assay (Figure 2a). Binding increased significantly from round 12 to round 14 in later stages of the SELEX with round 14 tentatively being suspected to be sufficiently specific for the intended application to serve as a polyclonal library of affinity molecules for specific labeling of *P. distasonis* cells and detection of this strain in complex mixtures of gut bacteria. The specificity of the polyclonal aptamer library for the surface targets of *P. distasonis* cells was then inspected for each round between 12–14 in a discrimination assay, where an ensemble of five other abundant gut bacteria including *A. muciniphila*, *A. stercoricanis*, *B. producta*, *R. microfusus*, and *R. intestinalis* were used as negative controls. The ability of the respective aptamer libraries to specifically label and discriminate *P. distasonis* from the control strains increased significantly with the labeling efficiency from round 12 to round 14 with the final library delivering fluorescence signals exclusively for *P. distasonis* (Figure 2b). This capability of the R14 library to discriminate *P. distasonis* from individual control bacteria was verified by labeling experiments of the same by measuring the fluorescence intensity of the aptamer bound bacterial strains for subsequent analysis by fluorescence microscopy. Labeling was only achieved with *P. distasonis*, whereas R14 completely failed to label the control bacteria (Figure 2c). This not only proved the specificity of R14 towards the dedicated target strain, but also demonstrated that this polyclonal library was immediately suited for labeling of its target for microscopical analysis, without the need of further efforts for identification of individual aptamers, which was earlier also observed for the FluCell-SELEX derived libraries against human-pathogenic and carbapenem-resistant *Pseudomonas aeruginosa* strains, which in addition were found to even outperform individual aptamers with respect to the robustness of target identification and the efficient labeling fidelity of different clinical isolates [13]. Here, clear fluorescence signals were exclusively observed for *P. distasonis* and collocated with the cells as expected. In contrast, the control bacteria were not fluorescently labeled demonstrating the specificity of the R14 library (Figure 2c).

**Figure 3:**
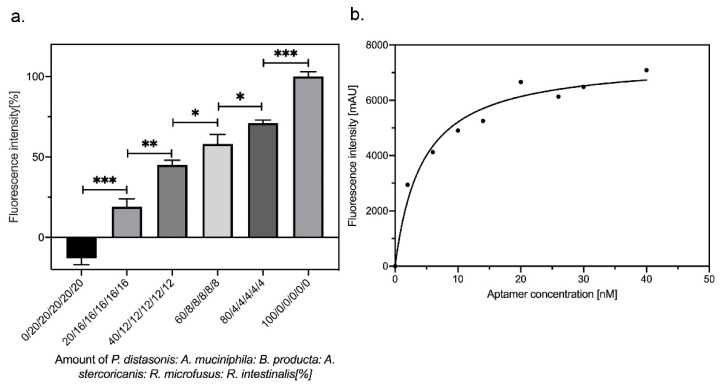
The polyclonal aptamer library R14 can specifically detect *P. distasonis*. (**a**) Detection of increasing amounts of *P. distasonis* by a Cy5-labeled polyclonal aptamer library R14 in a mixture of gut bacteria *B. producta, A. muciniphila, R. microfusus, A. stercoricanis,* and *R. intestinalis*, which were adjusted in equal optical densities and mixed at different ratios; *p-*values < 0.05 were considered significant; * denotes *p* < 0.05; ** *p* < 0.01 and *** *p* < 0.001. (**b**) One site-specific binding for polyclonal aptamer library R14. *P. distasonis* cells were incubated with different concentrations of the aptamer library R14, the resulting curve was fitted by nonlinear regression using the one site-specific binding model in GraphPad Prism 8. The polyclonal aptamer library R14 dissociation constant was calculated as 4.3 nM, and the deviation of the coefficient of determination was 0.9714.

The specificity of the R14 suggested, that this focused library might already be suited for retracing of given numbers of the target strain *P. distasonis* in mixtures with the control subset of gut bacteria as “contaminations” thereby allowing semi-quantitative analysis of increasing amounts of *P. distasonis* cells in these model bacterial communities. The ratios of control bacteria and *P. distasonis* were reduced from 0:1 (*P. distasonis*:controls strains) to 1:0 (*P. distasonis*:control strains). Labeling with the Cy5 R14 library and subsequent fluorescence measurements revealed that the increasing amount of *P. distasonis* along this series of samples could perfectly be followed and showed a linear relationship with sufficient or high significance levels (Figure 3a). When fixed amounts of *P. distasonis* cells were incubated with different and increasing aptamer concentrations increasing fluorescence intensities could be measured as expected and the curve fitted with a typical one site-specific binding model (Figure 3b). The deviation of the coefficient of determination R^2^ was 0.9714 and the calculated the low dissociation constant (K_d_: 4.3 nM) of the polyclonal aptamer library R14 further proved its high affinity for *P. distasonis* (Figure 3b) which also provided a theoretical basis for accurate and sensitive detection of *P. distasonis* [32].

## 4. Discussion

Since SELEX based procedures for the evolution of aptamers against epitopes on cells were first developed by Daniels et al. in 2003, new cell screening variants have been continuously developed as improvements of the methodology [27]. Namely, the TECS SELEX [33], FACS-SELEX [34], 3D cell SELEX [35] and cell-internalization SELEX [36] are known examples for these elaborated techniques. Recently we have demonstrated with the FluCell-SELEX the ease of a fluorescence-based cell screening method that allows efficient monitoring of the binding of aptamers to target cells simply based on the intensity of the fluorescence signal [13]. Moreover, the task to identify pathogenic *P. aeruoginosa* was could perfectly accomplished using already using the polyclonal FluCell-SELEX library or even better than using the best individual aptamers isolated from this library [13]. Here, the polyclonal library evolved by the same FluCell-SELEX method and directed against the potential probiotic gut bacterium *P. distasonis* proved this concept as the library could specifically label the dedicated *P. distasonis* target cells and allowed to retrace different amounts in mixtures with other bacteria with high abundance in gut microbiomes. Moreover, binding was demonstrated to happen with high affinity as demonstrated by the overall K_d_-value in the low nM range. 

Biosensor technologies in general are of still emerging importance for applications in biomedical diagnosis as well as in a wide range of other fields. Immediate monitoring of disease progression, drug discovery, and biomedical research are typical applications [12]. In 1956, Leland C. Clark Jr. successfully developed the first “real” biosensor for oxygen detection. Currently the database ‘Pubmed’ has indexed over 53,000 reports on the topic of ‘biosensors’ in the past decade. Most current biosensors fall into four main categories: bioaffinity, catalysis, transmembrane, and cellular sensors, with the probably most important active parts being based on catalytic principles or so called bioaffinity [37]. Thus, bioaffinity per se has a pivotal role as a key construction and functionality element in the development of biosensors, which is mainly still realized by the use antibodies or antibody derivatives [15]. However, this role of affinity molecules can be taken over with success by aptamers [38,39].

We believe that polyclonal aptamer libraries will revolutionize the field of biosensors due to their potentially excellent performance. Since the COVID-19 outbreak in 2020, humankind was forced to improve awareness of global crises posed by epidemics [40]. A key to success in handling and damming pandemics is how fast diagnostic tools, vaccines and therapeutics can be developed. The use of aptamers evolved by SELEX processes and especially the direct use of polyclonal libraries without extensive selection of single binders and development of individual aptamers may represent a considerable possibility to isolate evolve potent binding entities for different diagnostic assays in the management of epidemics in the future, as the development of such libraries is extremely fast. Based on the *P. distasonis* library presented here our next aim is to develop novel optical and electrical sensors (aptasensors) as new clinical diagnostic tools for the rapid detection of intestinal bacterial abundance. Therefore, one of the most common aptasensors is the duplexed aptamer and in terms of chip-based technology, electrochemical genosensors have been recently developed opening routes to the construction of microfluidic lab-on chis for highly efficient and specific biosensing [41,42,43]. As there is increasing evidence that gut bacteria are associated with many diseases such as obesity, diabetes, liver disease, cancer, and some neurodegenerative diseases. Therefore, human gut bacteria have now been considered as a source of novel potential therapies [44,45,46,47]. Specific aptamer-based biochips thus may open new avenues for the rapid detection of intestinal bacterial abundance and to monitor the success of future supplementation strategies involving promising intestinal probiotics like *P. distasonis* and other gut bacteria, which are currently developed in laboratories worldwide to also enhance human immunity against infections and to prevent diseases such as liver disease and neurodegenerative disorders.

## Supplementary Materials

The following are available online at https://www.mdpi.com/article/10.3390/microorganisms9112284/s1, Table S1: title. The selection condition. The amount of aptamer library, counter SELEX, target SELEX, incubation temperature and incubation time, washing condition, and the amount of BSA/tRNA are indicated here.
