# Supplementary material for: Polyclonal Aptamers for Specific Fluorescence Labeling and Quantification of the Health Relevant Human Gut Bacterium Parabacteroides distasonis
Source: Microorganisms. 2021 Nov 2;9(11):2284. doi: 10.3390/microorganisms9112284 (PMC8618460; doi:10.3390/microorganisms9112284)
Supplement: Supplementary file 1 [file microorganisms-09-02284-s001.zip › microorganisms-1419916-supplementary.pdf]

Supplementary Materials

Table S1. The selection condition. The amount of aptamer library, counter SELEX, target SELEX, incubation temperature and incubation time, washing condition, and the amount of BSA/tRNA were indicated here.

SELEX rounds	Aptamer (pmol)	Counter SELEX (Incubation temperature and time)	Target SELEX (Incubation temperature and time）	Wash times	BSA/tRNA (pmol)
1	500	-	250 μL OD600=1<br>Incubation：37 °C for 1 h	1	600
2	10	-	250 μL OD600=1<br>Incubation：37 °C for 1 h	1	900
3	10	-	250 μL OD600=1<br>Incubation：37 °C for 1 h	2	1200
4	10	-	250 μL OD600=1<br>Incubation：37 °C for 1 h	2	1500
5	10	-	250 μL OD600=1<br>Incubation：37 °C for 1 h	3	1800
6	10	250 μL OD600=2 *R. microfusus*<br>Incubation：37 °C for 1 h	250 μL OD600=1<br>Incubation：37 °C for 1 h	4	2100
7	10	50 μL OD600=2 per bacterium<br>Incubation：37 °C for 1 h	250 μL OD600=1<br>Incubation：37 °C for 1 h	6	2400
8	10	50 μL OD600=2 per bacterium<br>Incubation：37 °C for 1 h	250 μL OD600=1<br>Incubation：37 °C for 1 h	6	2700
9	10	50 μL OD600=2 per bacterium<br>Incubation：37 °C for 1 h	250 μL OD600=1<br>Incubation：37 °C for 1 h	6	3300
10	10	50 μL OD600=2 per bacterium<br>Incubation：37 °C for 1 h	250 μL OD600=1<br>Incubation：37 °C for 1 h	6	3600
11	10	50 μL OD600=2 per bacterium<br>Incubation：37 °C for 1 h	250 μL OD600=1<br>Incubation：37 °C for 1 h	6	3900
12	5	50 μL OD600=2 per bacterium<br>Incubation：37 °C for 1 h	250 μL OD600=1<br>Incubation：37 °C for 1 h	6	4200
13	5	50 μL OD600=2 per bacterium<br>Incubation：37 °C for 1 h	250 μL OD600=1<br>Incubation：37 °C for 0.5 h	6	4500
14	0.5	500 μL OD600=2 per bacterium<br>Incubation：37 °C for 1 h	250 μL OD600=1<br>Incubation：37 °C for 0.5 h	6	4500

Note: 1. To avoid non-specific interaction between the aptamer and the cell surface, the BSA (100 mg/mL) and tRNA (10 mg/mL) as competitors were incubated with targeted cells. 2. Counter-selection: Aptamer library was incubated with *A. muciniphila, A. stercoricanis, R. intestinalis, B. producta,* and *R. microfusus.* 3. Target SELEX: Aptamer library was incubated with *P. distasonis*.
